# cVEP Training Data Validation—Towards Optimal Training Set Composition from Multi-Day Data

**DOI:** 10.3390/brainsci12020234

**Published:** 2022-02-08

**Authors:** Piotr Stawicki, Ivan Volosyak

**Affiliations:** Faculty of Technology and Bionics, Rhine-Waal University of Applied Sciences, 47533 Kleve, Germany; piotr.stawicki@hochschule-rhein-waal.de

**Keywords:** brain–computer interface (BCI), code-modulated visual evoked potentials (cVEP), BCI speller, task-related component analysis (TRCA), canonical correlation analysis (CCA)

## Abstract

This paper investigates the effects of the repetitive block-wise training process on the classification accuracy for a code-modulated visual evoked potentials (cVEP)-based brain–computer interface (BCI). The cVEP-based BCIs are popular thanks to their autocorrelation feature. The cVEP-based stimuli are generated by a specific code pattern, usually the m-sequence, which is phase-shifted between the individual targets. Typically, the cVEP classification requires a subject-specific template (individually created from the user’s own pre-recorded EEG responses to the same stimulus target), which is compared to the incoming electroencephalography (EEG) data, using the correlation algorithms. The amount of the collected user training data determines the accuracy of the system. In this offline study, previously recorded EEG data collected during an online experiment with 10 participants from multiple sessions were used. A template matching target identification, with similar models as the task-related component analysis (TRCA), was used for target classification. The spatial filter was generated by the canonical correlation analysis (CCA). When comparing the training models from one session with the same session’s data (intra-session) and the model from one session with the data from the other session (inter-session), the accuracies were (94.84%, 94.53%) and (76.67%, 77.34%) for intra-sessions and inter-sessions, respectively. In order to investigate the most reliable configuration for accurate classification, the training data blocks from different sessions (days) were compared interchangeably. In the best training set composition, the participants achieved an average accuracy of 82.66% for models based only on two training blocks from two different sessions. Similarly, at least five blocks were necessary for the average accuracy to exceed 90%. The presented method can further improve cVEP-based BCI performance by reusing previously recorded training data.

## 1. Introduction

The electroencephalography (EEG)-based brain–computer interface (BCI) is a technology that has the potential of replacing, enhancing, and improving human interaction with the surrounding/environment as well as enhancing digital life [1,2,3,4].

While the most popular and easy to implement paradigms are based on the visually evoked potentials (e.g., SSVEP-based BCI [5,6]), the code-modulated VEP (cVEP) has been highly researched in the recent past with very promising results in terms of accuracy and information transfer rate (ITR) [7,8]. Spüler et al. used a one class support vector machine and error-related potentials for an online adaptation of a cVEP-based BCI system [9]. In 2016, Nakanishi et al. utilised canonical correlation analysis (CCA) and datasets recorded on two different days to evaluate the performance of different session-to-session transfer learning approaches, in order to optimise the SSVEP classification [10]. Nakanishi et al. presented an enhanced ensemble task-related component analysis (TRCA) method, where the spatial filter created from different targets were combined in order to increase the overall SSVEP-based BCI system performance [11]. In an online cue-guided experiment with 20 subjects, a mean accuracy of 89.8% was achieved. In another relevant paper published in 2015, Yuan et al. improved the performances of an SSVEP-based BCI while exploiting inter-subject template transformation [12]. Compared to the standard CCA, when using the proposed method, readings of 7.4% and 18.8% increases in accuracy for data lengths (time-window lengths) of 2.0 s and 1.5 s, respectively, were obtained for 12 participants in a simulated online experiment. Wong et al. presented a subject transfer-based CCA method to combine the information between the subjects and within the subject, reaching an average ITR of 198.18 bits/min [13]. Wang et al. proposed an inter- and intra-subject template-based multivariate synchronization index with an adaptive threshold for a 12-class SSVEP-based BCI dataset [14]. The results from 10 subjects showed an average accuracy of 99.2% for the new method compared to the standard CCA, which reached 93.6%. In 2020, Gembler et al. presented a session-to-session training transfer for a cVEP-based BCI system [15]. Eight out of 10 participants achieved an average accuracy of 97.1% when utilising the training data recorded 2 weeks earlier in an online copy-spelling task.

Exploring inter-subject variability is recently a popular research interest in the BCI field across different paradigms: in the sensorimotor rhythms (SMR)-based BCI system e.g., Saha et al. [16], Saha and Baumert [17]; in the simultaneous P300 event-related potential (ERP) and functional near-infrared spectroscopy (fNIRS) by Li et al. [18]; and in SSVEP-based BCI, Wei et al. [19], Tanaka [20]. In a recent review, Zarefa et al. compared different approaches to the users’ training and feature extraction algorithms of SSVEP-based BCI systems [21].

In 2021, Stawicki et al. [22] tested similarities in SSVEP and steady-state motion visual evoked potentials (SSMVEP) training by cross-analysing the training sessions for SSVEP and SSMVEP stimulus designs. The analysed data came from a previously performed comprehensive study with 86 participants [8].

The common necessity is that long repetitive training sessions ensure good signal-to-noise ratios for VEP-based BCI analysis. However, with respect to user-friendliness, these commonly-used repetitive training sessions may be tedious, as subjects are required to focus their gaze for a relatively long time on the stimulus, usually, multiple times. This is a time-consuming approach that commonly causes visual fatigue [8]. While the BCI system was developed for everyday use, alternative approaches were beneficial, such as training-free algorithms or subject-independent methods. Recent popular approaches in the SSVEP-based BCI systems are the transfer of subject-specific learning across stimulus frequencies [23], by transferring the learning knowledge within the subject and between subjects [24], or transferring training data between sessions [15].

Recently developed novel cVEP-based BCI systems focus on skipping the mostly required training session [25,26]. In 2018, Nagel and Spüler developed a model that predicts the arbitrary visual stimuli from EEG and the triggered brain response [25]. In an online experiment, nine participants achieved an average accuracy and ITR of 98.0% and 108.1 bits/min, respectively, for an optimised EEG2Code model using 32 EEG electrodes. In the recent paper, Thielen et al. developed a calibration-free method (0-training approach), where in an online copy-spelling task, nine participants achieved an average accuracy and ITR of 99.7% and 67.9 bits/min, respectively, using eight EEG electrodes and gold codes-based stimuli [26].

The herein paper utilises previously recorded EEG data from a cVEP study with 10 subjects [15]. The recordings were analysed with transfer data from one session to another session (both directions) of the same participant, and transfer data between different participants and sessions were included. In other words, the models (training data) from one participant were used to classify the data (testing data) from all participants from the same session and for the testing data of all participants of the other session. During this study (further referred to as the original study), two recording sessions took place per participant, which were spread apart by about 2 weeks. Here, we combine these recorded data into one pool, which is further analysed.

## 2. Materials and Methods

### 2.1. Participants

The data of the 10 participants of the original experiment [15] were utilised in this offline analysis. All subjects were recruited from Rhine-Waal University of Applied Sciences (two female), with a mean (SD) age of 25.4 years (4.1). A written informed consent was signed before the experiment; the experiment was approved by the ethical committee of the medical faculty of the University Duisburg-Essen, Germany. Participants had normal or corrected-to-normal vision and little to no prior BCI experience. All participants received a financial reward for their participation.

### 2.2. Stimulus Presentation

**Figure 1:**
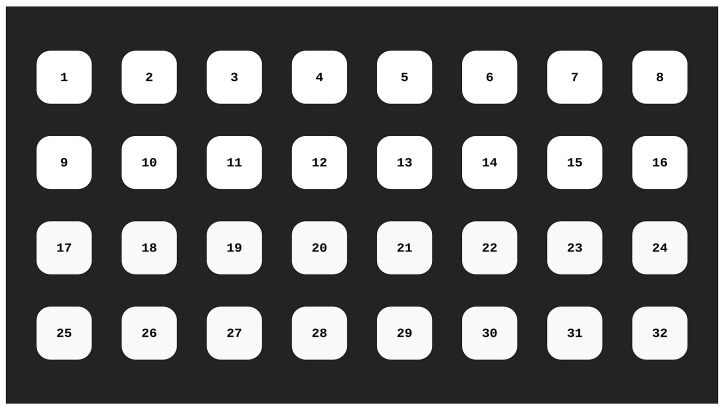
The graphical user interface (GUI) used in the training phase of online experiment [15] with 32 targets. During the training session, the current target on which the participants needed to fix their gaze was marked with a green frame.

The stimuli were presented on a 24.5-inch monitor (Acer predator XB252Q) with a refresh rate of 240 Hz and a resolution of 1920 × 1080 pixels, which was positioned approximately 60 cm from the subject’s eyes [15]. The stimuli consisted of 32 white-squared targets with sizes of 150×150 pixels or 4.3×4.3 cm, which correspond to a visual angle of 4.1°, presented over a black background; see Figure 1. The distance between the targets was 75 pixels or 2.1 cm (vertical and horizontal).

The cVEP stimuli used the 63-bit m-sequence $c_{1}$ = 101011001101110110100100111000101111001010001100001000001111110 [27], where ‘0’ represented ‘black’ and ‘1’ represented ‘white’, thus presenting at full contrast. The remaining stimuli $c_{k},k=2,\cdots ,K$, where K = 32, were generated by circularly left shifting $c_{1}$ by k·2 bits. The update rate was set to 60 Hz; thus, the colour of the stimulus changed in accordance with the bit sequence: every fourth frame with the used refresh rate of 240 Hz. This m-sequence was displayed for a duration of 1.05 s, which emulates to the historical vertical refresh rate of 60 Hz, which was popular in many previous studies.

In order to achieve the most reliable stimulus presentation, the G-Sync^®^ technology was disabled. Instead, the fixed refresh rate (240 Hz) was used, where a single frame is drawn within 4.17 milliseconds. Additionally, with the graphic card manufacturer tool, the number of pre-rendered frames (in the graphics card memory) was set to 1 (minimal available value). This reduced the internal drawing delays usually present in graphics card hardware to the actual drawing on the screen during the vertical screen refresh. Further details regarding the stimulus design can be found in [15].

### 2.3. Recordings

The recordings used for this study were the training phase of the original study, which consisted of a series of six repetitions (blocks). In every block, the participants had to focus their gaze at each target, which was cued sequentially through all 32 targets, for 2.10 s with a gaze shift pause of 1.0 s between every target. Between the blocks, the participants had an opportunity for a short break (while the EEG was still connected and the participant was still seated), after which they continued by pressing the space key.

### 2.4. Hardware and Software for Data Analysis

The hardware used for the offline analysis was a MSI GT73 notebook equipped with an Intel processor (Intel Core i7-6820HK CPU @2.70 GHz) and 16 GB of RAM, running on Windows 10 Education. The software utilised for the offline analysis was MATLAB^®^ 2021b, and the ensemble TRCA methods from [11] were adopted.

### 2.5. CCA-Based Spatial Filter Design and Template Generation

Canonical correlation analysis is the most popular and widely used method for generating the spatial filters, which finds a linear transformation that maximizes the correlation between the recorded signal and a template signal, e.g., sine–cosine signals or averaged EEG template signals [28,29]. Typically, only the first canonical correlation and corresponding weights (w) are used for the classification and construction of filters [11].

Similar to our previous study, we combined the CCA method with the TRCA template construction [15,22,30]. Each training trial was stored in an m×n matrix, where *m* denotes the number of electrode channels (here, m=16) and *n* denotes the number of sample points (here, there are two 1.05 s stimulus cycles, $n=1.05\cdot F_{S}\cdot 2=1260$).

Given two multi-dimensional variables $X\in R^{m_{1}\times n}$ and $Y\in R^{m_{2}\times n}$, CCA identifies weights $w_{X}\in R^{m_{1}}$ and $w_{Y}\in R^{m_{2}}$ that maximize the correlation, ρ, between the so-called canonical variates $x=X^{T}w_{X}$ and $y=Y^{T}w_{Y}$ by solving

(1) $$\rho =\max_{w_{X},w_{Y}}\frac{{w_{X}}^{T}XY^{T}w_{Y}}{\sqrt{{w_{X}}^{T}XX^{T}w_{X}\;{w_{Y}}^{T}YY^{T}w_{Y}}}.$$

The correlation value ρ that solves (1) is the canonical correlation. For most VEP-based BCI realizations, only the first canonical correlation weights ($w_{X}$, $w_{Y}$) are used for classification or for the spatial filter design.

The recorded data were segmented into single trials $T_{i}\in R^{m\times n}$, and used to generate a CCA-based spatial filter $w\in R^{m}$. In total, 32×6=192 of such trials $T_{i,j}\in R^{m\times n}$, i=1,…,K, $j=1,\ldots ,n_{b}$, where $n_{b}=6$, were recorded.

For each target, individual templates $X_{i}\in R^{m\times n}$ and filters $w_{i}$ were determined (i=1,…,K). In order to generate the spatial filters, the two matrices were constructed,

(2) $${\hat{T}}_{i}=\left[T_{i,1}T_{i,2}\ldots T_{i,n_{b}}\right]\;\mathrm{and}\;X_{i}=\left[\underset{n_{b}}{\underset{︸}{{\hat{X}}_{i}{\hat{X}}_{i}\ldots {\hat{X}}_{i}}}\right],$$

where ${\hat{X}}_{i}$ represents the average of the $n_{b}$ trials corresponding to the *i*-th class. When inserted into Equation (1), these two matrices yield the filter vectors: $w_{i}=w_{{\hat{T}}_{i}}$, i=1,…,K.

### 2.6. Classification

In order to add further improvements to the target classification, the ensemble spatial filter *w*, introduced in the TRCA method [11], which concatenates the spatial filters of all targets (classes), and the filter bank-based analysis [31], were implemented. For the filter bank design, $a_{k}$ was calculated as

(3) $$a_{k}=\frac{\alpha_{k}}{\sum_{k=1}^{K}\alpha_{k}},\mathrm{where}\;\;\;\alpha_{k}=k^{-1.25}+0.25,$$

which gives the following normalised results: 0.386, 0.207, 0.156, 0.132, and 0.119. The lower cut-off frequencies for the *k*-th sub-band were 6, 14, 22, 30, and 38 Hz, and the upper cut-off frequency was 60 Hz for all-sub, which was filtered with an 4th-order Butterworth filter. In order to cancel out any phase response, forward and reverse filtering methods were applied.

The output command (*C*) was calculated based on the weighted linear combinations of the Pearson’s correlation coefficient ($\rho_{k}\left({\hat{X_{i}}}^{T}w_{i},{\hat{T_{i}}}^{T}w_{i}\right)$) and the filter bank sub-bands (*K*) and amplitude ($a_{k}$),

(4) $$C=\underset{k=1,\ldots ,K}{arg max}\lambda_{k}\;,\;\mathrm{where}\;\lambda_{K}=\sum_{k=1}^{K}a_{k}\rho_{k}$$

for every trial in each block.

The information transfer rate was calculated utilising the regular ITR formula:

(5) $$ITR=(log_{2}\left(N\right)+Plog_{2}\left(P\right)+\left(1-P\right)log_{2}\left(\frac{1-P}{N-1}\right))*60/T,$$

where *N* is the number of targets, *P* is accuracy, and *T* is the total time, including the gaze shift (inter-trial) pause. An online ITR calculator can be found at (https://bci-lab.hochschule-rhein-waal.de/en/itr.html, accessed on 31 December 2021).

### 2.7. Procedure

**Figure 2:**
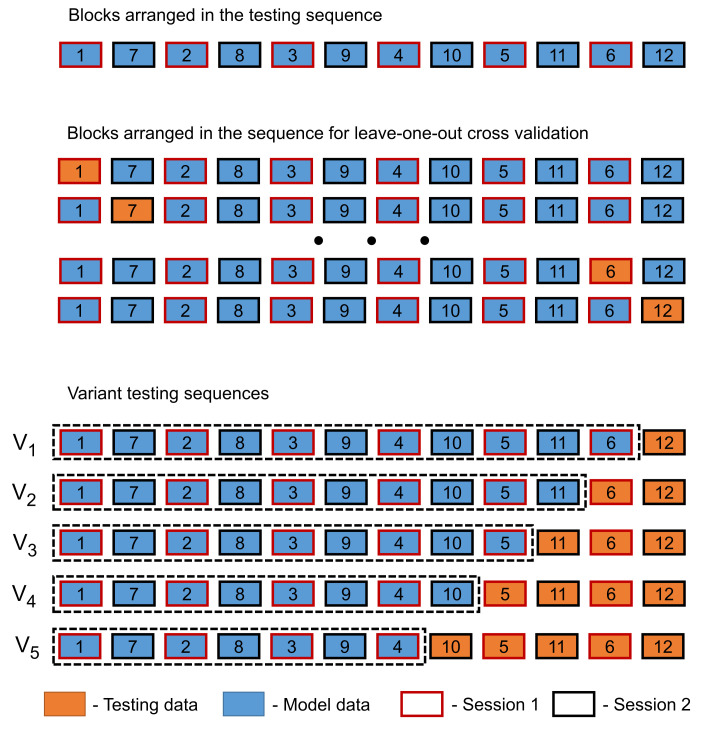
Different testing arrangements used in this study. The training data from the original experiment [15] were divided into 12 blocks and rearranged into a testing sequence, which was divided into model building and testing datasets.

Both training sessions from the original study [15] were recorded on different days (mean distance 14.4 days), where each training session contained 6 training blocks. All the blocks from both sessions were used as follows: the testing data from 1st session ($D_{1}$) blocks were numbered from 1 to 6, and the testing data from 2nd session ($D_{2}$) blocks were numbered 7 to 12; see Figure 2.

For this study, the blocks were arranged in the following sequence (seq) = 1, 7, 2, 8, 3, 9, 4, 10, 5, 11, 6, and 12 for the leave-one-out, stepwise correlation and for 5 different variant combinations, which are described as follows (see Figure 2):

Variant 1 ($V_{1}$), where block bl12 was used for testing and blocks bl1–bl11 in seq order were used for model building.
Variant 2 ($V_{2}$), where blocks bl12andbl6 were used for testing and blocks bl1–bl11 in seq order without bl6 were used for model building.
Variant 3 ($V_{3}$), where blocks bl12,bl6,andbl11 were used for testing and blocks bl1–bl10 in seq order without bl6 were used for model building.
Variant 4 ($V_{4}$), where blocks bl12,bl6,bl11,andbl5 were used for testing and blocks bl1–bl10 in seq order without bl5 and bl6 were used for model building.
Variant 5 ($V_{5}$), where blocks bl12,bl6,bl11,bl5,andbl10 were used for testing and blocks bl1–bl9 in seq order without bl5 and bl6 were used for model building.

**Figure 3:**
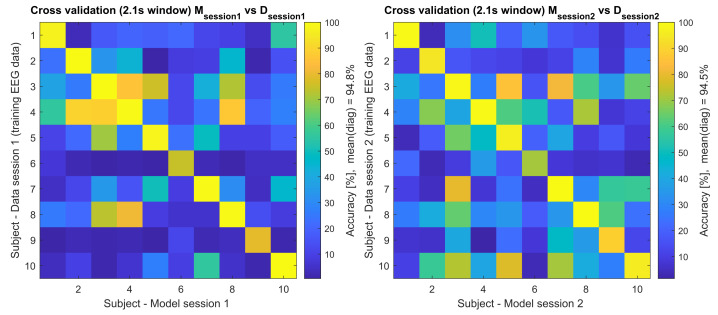
Cross-validation of the training EEG data (2.1 s). This figure shows the accuracy of M${}_{\mathrm{session}1}$Data${}_{\mathrm{session}1}$ and M${}_{\mathrm{session}2}$Data${}_{\mathrm{session}2}$ tested across all participants, where e.g., the model (M) from session 1 was used to classify the session 1 training EEG data (**left** side), and model (M) from session 2 was used to classify the session 2 training EEG data (**right** side).

**Figure 4:**
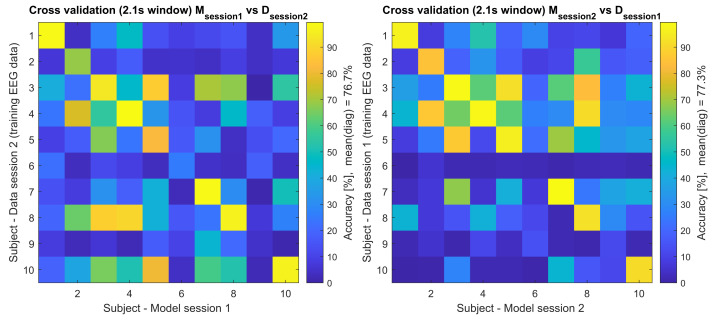
Cross-validation of the training EEG data across paradigms for 2.1 s time window. This figure shows the accuracy of M${}_{\mathrm{session}1}$Data${}_{\mathrm{session}2}$ and M${}_{\mathrm{session}2}$Data${}_{\mathrm{session}1}$ tested across all participants, where e.g., the model (M) from session 1 was used to classify the session 2 training EEG data (**left** side), and model (M) from session 2 was used to classify the session 1 training EEG data (**right** side).

Additionally, a cross testing between session 1 ($D_{1}$) containing bl1–bl6 and session2 ($D_{2}$) containing bl7–bl12 was performed, where models built from one session were used for testing the data from the other session, e.g., model M (training data, x-axis) from subject 1 session 1 was used to classify the testing data (y-axis) of all subjects from the same session (session 1, Figure 3, left side) and all subjects from the other session (session 2, Figure 4, left side). The other direction was also analysed e.g., model M (training data, x-axis) from subject 1 session 2 was used to classify the testing data (y-axis) of all subjects from the same session (session 2, Figure 3, right side) and all subjects from the other session (session 1, Figure 4, right side). The accuracy of these results is presented in Figure 3 and Figure 4, and the detailed diagonals are presented in Table 1.

The stepwise increasing concatenated models were built as follows (e.g., for testing bl12):

- Step 1—model bl1
- Step 2—model bl1+bl7
- Step 3—model bl1+bl7+bl2
- Step 4—model bl1+bl7+bl2+bl8
- Step 5—model bl1+bl7+bl2+bl8+bl3
- Step 6—model bl1+bl7+bl2+bl8+bl3+bl9
- Step 7—model bl1+bl7+bl2+bl8+bl3+bl9+bl4
- Step 8—model bl1+bl7+bl2+bl8+bl3+bl9+bl4+bl10
- Step 9—model bl1+bl7+bl2+bl8+bl3+bl9+bl4+bl10+bl5
- Step 10—model bl1+bl7+bl2+bl8+bl3+bl9+bl4+bl10+bl5+bl11
- Step 11—model bl1+bl7+bl2+bl8+bl3+bl9+bl4+bl10+bl5+bl11+bl6

For the leave-one-out cross-validation, if one block was tested, the steps would be built accordingly without these blocks.

The accuracy was calculated for every trial out of 32 in one block.

## 3. Results

Table 1 shows the results for session 1 (bl1–bl6) vs. session 2 (bl7–bl12) cross analysis, where the training data of one session were used to create the reference models *M* and the data from both were used for testing *D*. The training data of session 1 was used to build the model session 1 ($M_{session1}$), and this model was used to classify the testing data from session 1 ($D_{session1}$) and the testing data of session 2 ($D_{session2}$). Similar, the training data of session 2 was used to build the model session 2 ($M_{session2}$), and this model was used to classify the testing data of sessions 1 ($D_{session1}$) and session 2 ($D_{session2}$). A pairwise Mann–Whitney U-test between the $M_{session1}D_{session1}$ vs. $M_{session1}D_{session2}$ showed a statistical significance with U = 21.5, Z = −2.17, *p* < 0.05; between the $M_{session1}D_{session1}$ vs. $M_{session2}D_{session1}$ showed a statistical significance with U = 23.0, Z = −2.05, *p* < 0.05; between the $M_{session2}D_{session2}$ vs. $M_{session1}D_{session2}$ showed no statistical significance with U = 30.5, Z = −1.48, *p* = 0.14; between the $M_{session2}D_{session2}$ vs. $M_{session2}D_{session1}$ showed no statistical significance with U = 30.5, Z = −1.48, *p* = 0.14.

The bottom part of Table 1 shows the corresponding information transfer rates (ITRs), as shown in Equation (5).

The values in Table 1 also represent the corresponding diagonals of Figure 3 and Figure 4, where the additional cross-subject validation was performed. In Figure 3, the models and data from the same session (intra session) were cross-validated between the subjects and in Figure 4 the models and data from the other session (inter session) were cross-validated between the subjects.

**Figure 5:**
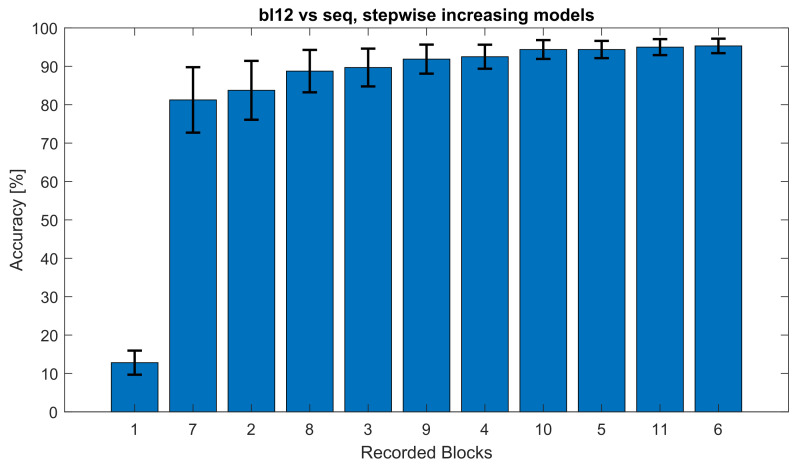
This figure shows the classification accuracy of the bl12 against models built from all other blocks arranged in the testing sequence (seq) = 1, 7, 2, 8, 3, 9, 4, 10, 5, 11, 6. The models were built stepwise added together; the blocks in the seq order, e.g., “1”, “7”, “2”, “8”, etc. represent the models built from bl1, bl1+bl7, bl1+bl7+bl2, and bl1+bl7+bl2+bl8, respectively. The whiskers mark the standard error.

In order to test the interchangeability of single training blocks from different multi-day sessions, the testing sequence arrangement (see Figure 2) was constructed and evaluated in Figure 5 with a single block *bl12* against a concatenating increasing model from both sessions. This approach was also used to compare all other block from the multi-day pool in a leave-one-out cross-validation with a stepwise increasing (concatenating) models, including all other blocks in Table 2. The eleven bars in Figure 5 corresponds to the column labelled *12* in Table 2.

The different variants constructed in Figure 2 and evaluated in Table 3 test the different multi-day training data in order to build a minimal training model for improving the accuracy. Here, a Kruskal–Wallis Test showed no statistical significance between the different variants $V_{1}$–$V_{5}$ with H(4) = 0.271, *p* = 0.992.

## 4. Discussion

This paper focuses on the offline analysis of the cVEP training data only of the original study [15]; the training sessions were unified for all participants of the original study, which was performed on 2 separate days, spread apart for around 14 days.

Here, the focus lies on finding the suitable training process for the used cVEP system by cross-analysing the training data of both sessions and finding the best training strategy. First, the training data of each session were used for TRCA model building and testing (see Figure 3). When analysing this training intra-session’s classification accuracies, averaged over all six blocks of a leave-one-out cross-validation, session 1 and session 2 reached 94.84% and 94.53%, respectively, which correspond to an ITR of 88.0 bits/min and 87.1 bits/min, respectively (see Table 1).

Both training sessions’ data from the 1st and the 2nd day, session 1 and session 2, respectively, were cross-validated against the models from the other session (inter-session); see Table 1 and Figure 4. Interestingly, this cross-session analysis (presented in Table 1) shows that the participants could still achieve reliable control over the system with accuracies at around 76.67% and 77.34%, which shows that the fresh model can classify old data.

In order to avoid favouring the second session and the dependencies arising from the session continuity, the blocks data of both sessions were interchangeably (alternately) stacked, creating a continuous testing sequence seq. This sequence was also used for stepwise increasing models. These models were analysed with a leave-one-out cross-validation of every block and averaged across participants (Table 2), yielding a maximum accuracy of 96.88%, which is higher than the intra-session average of both of the sessions (Table 1). This result was achieved for testing the block bl8 with the model at the step 6. The results of the testing sequence show a relevant increase in accuracy when additional blocks are added (increasing step number) to the model build. Interestingly, in step 2, where just two blocks were used for the model, the averaged accuracy was 82.66%. This accuracy increased with every additional step until it reached 94.44%.

Lastly, to find the minimalistic training procedure, the tested sequence seq data were arranged into five variants. In every variant, the accuracy was calculated by averaging the results for every testing block (see Table 3, Figure 2) with a fixed model. When analysing these results, the average accuracy had almost no difference between the variants $V_{1}$–$V_{5}$. Interestingly, when more testing data were used in a variant (e.g., $V_{5}$), there was only a slight increase in the classification accuracies, reaching 93.56% when compared to e.g., $V_{2}$, which achieved 92.5%, the same as $V_{1}$.

These results show that from at least two sessions a minimum of two training blocks are required, in order to achieve sufficient cVEP-based BCI system control, so that the accuracy exceeds 80%, as mentioned in Renton et al. [32]. In this case, the average classification accuracies achieved 82%, and if at least five training blocks are used, the average accuracy exceeds 90% (see Table 2). The 2nd step of the tested sequence included the 1st blocks of both sessions, thus, one old (previous session) and one new (current session) block of training data. The findings of this study suggest that the already collected training data can be efficiently reused, e.g., as transferred training data, to increase the user-friendliness of the BCI system by reducing repetitive training and thus minimising the potential visual fatigue caused by the stimuli.

Compared to the system developed by Thielen et al. [26], which only requires an initial warm-up period of 12 s, the here-presented approach requires a minimum of one new training block, which corresponds to 98.2 s (including the inter-trial time of 1.0 s). On the other hand, some minimal training can be beneficial (easier adaptation) for different stimulation hardware (higher refresh rates which corresponds to higher cVEP carrier frequency), where the number of gold codes can be limited; instead, longer m-sequences can be implemented: one example is a 124-bit quintary m-sequence [33]).

## 5. Conclusions

Our findings show that at least two training blocks from two sessions form a sufficient starting point for further improvements in a modern cVEP-based BCI system, which in future research can further be improved with e.g., an online adaptation process. While the novel developed/emerging systems can work without a user-specific training (e.g., Spüler et al. [9] or Thielen et al. [26]), most of the currently used and popular cVEP-based BCI systems require some training phase that could benefit from previously collected training data, also from other subjects. This cross-subject feature extraction requires further research. While nothing can replace a “fresh” EEG data from the training (a necessity for an optimal model), the need for new re-training data can be reduced to the bare minimum. This can help some potential users in re-using their own previously collected data for the BCI system for everyday use and thus boost the setup times and further increase the initial daily performance of a cVEP system. This is also valid for many other BCI paradigms.

## Figures and Tables

**Table 1:** Cross sessions accuracies (%) and ITR (bits/min) results. The data of session 1 (bl1–bl6) and session 2 (Dbl7–bl12) were used to create the models $M_{session1}$ and $M_{session2}$, respectively.

Accuracy	Model Session 1		Model Session 2	
Subject	Data Session 1	Data Session 2	Data Session 1	Data Session 2
1	97.40	98.96	96.88	99.48
2	100	68.75	84.90	94.27
3	100	94.27	98.96	98.96
4	99.48	99.48	98.44	99.48
5	98.44	82.29	96.35	96.88
6	75.00	25.52	02.08	70.83
7	99.48	98.96	99.48	100
8	99.48	96.88	92.71	100
9	79.17	04.69	11.98	89.06
10	100	96.88	91.67	96.35
Mean	94.84	76.67	77.34	94.53
SD	09.45	34.21	37.39	09.00
**ITR**	**Model Session 1**		**Model Session 2**	
**Subject**	**Data Session 1**	**Data Session 2**	**Data Session 1**	**Data Session 2**
1	90.91	94.16	89.9	95.37
2	96.77	49.47	70.44	85.15
3	96.77	85.15	94.16	94.16
4	95.37	95.37	93.03	95.37
5	93.03	66.75	88.9	89.90
6	57.10	09.50	00	51.95
7	95.37	94.16	95.37	96.77
8	95.37	89.90	82.49	96.77
9	62.51	00.10	02.14	76.64
10	96.77	89.90	80.78	88.90
Mean	88.00	67.45	69.72	87.10
SD	15.03	36.13	36.95	13.88

**Table 2:** Averaged accuracy (Acc.) results of the leave-one-out cross-validation of every single block of the testing sequence with the stepwise increasing model (step). Presented are means and SD (bottom table) of all 10 participants. The mean (SD) in the top part are average values for every step.

Acc.	Single Blocks of Testing Sequence													
Step	1	7	2	8	3	9	4	10	5	11	6	12	Mean	SD
1	13.44	13.13	19.69	15.63	21.25	11.88	18.44	12.50	22.81	10.63	16.56	12.81	15.73	3.35
2	83.13	75.00	83.75	89.69	83.44	82.81	82.81	84.06	84.69	79.69	81.56	81.25	82.66	2.19
3	85.63	86.25	86.25	90.31	86.25	85.94	87.19	89.38	85.94	82.81	87.50	83.75	86.43	1.44
4	88.13	89.06	88.13	90.94	88.44	89.38	89.38	91.56	87.81	87.50	85.94	88.75	88.75	1.09
5	89.06	90.31	88.44	95.31	88.13	90.00	92.81	90.94	90.63	88.44	88.44	89.69	90.18	1.51
6	90.31	92.19	90.00	96.88	90.31	90.00	92.50	92.81	92.50	91.56	88.13	91.88	91.59	1.54
7	90.31	93.75	90.63	95.94	90.94	92.81	93.13	94.69	94.38	92.50	90.63	92.50	92.68	1.43
8	93.13	93.75	92.81	95.63	93.13	92.81	93.75	94.69	93.44	92.81	90.94	94.38	93.44	0.83
9	93.13	94.06	91.88	95.94	93.75	93.75	95.00	95.31	94.06	92.81	91.25	94.38	93.78	1.02
10	93.44	94.06	94.38	96.25	93.13	93.44	96.25	94.06	95.63	92.50	91.56	95.00	94.14	1.13
11	93.13	94.38	93.13	96.56	92.81	94.06	96.25	95.31	96.25	93.44	92.50	95.31	94.43	1.26
**SD**	**Single Blocks of Testing Sequence**													
**Step**	**1**	**7**	**2**	**8**	**3**	**9**	**4**	**10**	**5**	**11**	**6**	**12**		
1	6.92	8.94	23.25	11.79	22.43	12.83	23.91	10.21	26.23	10.54	22.87	10.46		
2	33.79	34.86	32.93	18.81	31.84	29.4	29.4	27.02	28.81	34.37	31.06	28.41		
3	28.65	25.48	28.65	19.51	26.85	26.69	25.66	21.61	28.08	29.98	22.63	25.55		
4	24.15	19.78	24.77	18.72	22.44	19.72	22.59	18.58	26.49	22.92	25.1	18.41		
5	23.95	20.75	24.7	10.65	23.24	18.85	12.85	17.27	20.62	21.45	21.95	16.41		
6	21.52	17.26	19.7	6.91	19.62	19.08	13.6	15.8	17.07	16.01	21.13	12.6		
7	20.96	13.34	18.16	9.78	18.37	13.59	12.66	11.6	11.67	12.94	17.37	10.44		
8	15.22	13.34	14.44	10.74	14.49	13.74	11.41	13.59	13.3	13.01	16.76	8.18		
9	15.92	12.36	16.35	11.79	13.26	11.69	8.98	11.62	11.55	13.01	16.46	7.48		
10	13.78	12.36	11.1	10.81	14.49	12.45	6.56	12.8	7.68	12.94	15.45	6.94		
11	14.64	11.39	13.72	8.77	15.17	10.36	6.56	10.65	6.56	10.97	13.91	6.29		

**Table 3:** Accuracies of the different variants for the tested 10 participants (P1–P10).

Acc	$V_{1}$	$V_{2}$	$V_{3}$	$V_{4}$	$V_{5}$
P1	100	98.44	97.92	98.44	98.75
P2	93.75	98.44	96.88	97.66	98.13
P3	96.88	100	100	100	100
P4	100	98.44	98.96	98.44	98.75
P5	93.75	96.88	96.88	98.44	96.25
P6	71.88	70.31	68.75	68.75	66.25
P7	100	100	100	100	100
P8	100	98.44	98.96	99.22	99.38
P9	75.0	67.19	70.83	75.0	80.0
P10	93.75	96.88	96.88	97.66	98.13
Mean	92.50	92.500	92.61	93.36	93.56
SD	10.44	12.58	12.09	11.45	11.30
